# International spread or local outbreak? Epidemiologic analyses of transmission patterns of NDM-1-producing *Klebsiella pneumoniae* based on genomic surveillance data, Germany, January 2022 to February 2023

**DOI:** 10.2807/1560-7917.ES.2026.31.1.2500378

**Published:** 2026-01-08

**Authors:** Mirco Sandfort, Jessica Eisfeld, Jörg B Hans, Felix Reichert, Dunja Said, Martin A Fischer, Friederike Maechler, Brar Piening, Hanna Buck, Nadine Litzba, Torsten Semmler, Guido Werner, Tim Eckmanns, Niels Pfennigwerth, Sören Gatermann, Sebastian Haller, on behalf of the Generic IGS system development group

**Affiliations:** 1 Department of Infectious Disease Epidemiology, Robert Koch Institute, Berlin, Germany; 2 German National Reference Centre for Multidrug-resistant Gram-negative Bacteria, Department of Medical Microbiology, Ruhr-University Bochum, Bochum, Germany; 3 Department of Infectious Diseases, Robert Koch Institute, Wernigerode, Germany; 4 Institute of Hygiene and Environmental Medicine, Charité – Universitätsmedizin Berlin, Berlin, Germany; 5 National Reference Centre for Surveillance of Nosocomial Infections at the Institute of Hygiene and Environmental Medicine, Charité – Universitätsmedizin Berlin, Berlin, Germany; 6 Department MFI: Method Development, Research Infrastructure and Information Technology, Robert Koch Institute, Berlin, Germany; 7 The members of the study group are listed under Acknowledgements.

**Keywords:** Carbapenemase, Klebsiella pneumoniae, Ukraine, Disease Outbreaks, Multilocus Sequence Typing, Humans, Cross Infection, Communicable Disease Control

## Abstract

**BACKGROUND:**

Carbapenemase-producing Enterobacterales (CPE) cause infections, particularly nosocomially, with limited treatment options. NDM-1-producing *Klebsiella pneumoniae* cases have substantially increased since 2022, associated with the Ukraine war.

**AIM:**

We aimed to investigate transmission patterns using Germany’s Integrated Genomic Surveillance (IGS), combining notifications and sequence data.

**METHODS:**

We selected NDM-1-producing *K. pneumoniae* cases, confirmed by isolates between 1 January 2022 and 28 February 2023. Isolates were Illumina whole genome-sequenced and linked to notifications. Clusters were defined as ≤ 12 allelic differences in core genome-wide single nucleotide variant-based genotyping. Cluster categories were: ‘no exposure abroad’, ‘exposure in Ukraine’ or ‘other exposure abroad’ if ≥ one case stayed in Ukraine or elsewhere. Follow-up of 13 clusters examined further exposure information.

**RESULTS:**

Among 424 cases of most frequent sequence types, 326 (77%) belonged to 61 clusters. Seventeen (28%) clusters were associated with no exposure abroad, 33 (54%) with exposure in Ukraine, seven (11%) with other exposure abroad, and four (7%) had insufficient data. Cases in clusters with exposure in Ukraine were more dispersed, younger, and more often wound-infected than in other exposure location categories (p < 0.01). Cluster follow-up revealed one cluster with all cases from Ukraine or Russia, another with nosocomial transmission following case importation, and a third with all cases from one German hospital without exposure abroad.

**CONCLUSION:**

Most cases were in clusters, suggesting preventable chains of transmission. Three patterns emerged: transmission abroad, transmission in German hospitals from imported cases or local outbreaks. IGS can identify where transmission could be interrupted. International cooperation needs strengthening to prevent CPE spread.

**Key public health message**

**What did you want to address in this study and why?**

*Klebsiella pneumoniae* is a bacterial species that colonises the human gut and is transmitted in healthcare settings. It is of particular concern if resistant to antibiotics, e.g. carbapenems. Resistance can be conferred by carbapenemases such as NDM-1. Since 2022, NDM-1 *K. pneumoniae* has increased in Germany, partly associated with the war in Ukraine. We aimed to delineate where and how transmission occurred in 2022–23 to guide interventions.

**What have we learnt from this study?**

Most infections or colonisations with NDM-1 *K. pneumoniae* were linked in clusters, i.e. pathogens so genetically similar that transmission between cases or a similar source is suspected. The total number of clusters was higher than expected. We found three transmission patterns: transmission abroad before cases’ arrival and detection in Germany, transmission in Germany from imported cases, and local outbreaks in Germany.

**What are the implications of your findings for public health?**

Many cases could be attributed to chains of transmission, which is key information to better target public health measures to prevent further spread. A relevant proportion of cases in Germany likely stems from transmission abroad (particularly Ukraine). International cooperation should strengthen outbreak control in affected countries. Surveillance integrating genomic and epidemiologic data was able to detect and locate transmission.

## Introduction

The spread of antibiotic-resistant bacteria poses a major public health threat, as they cause severe, difficult-to-treat infections [1]. Bacteria of the order Enterobacterales colonise the gut and are prime candidates for healthcare-associated infections, which are especially concerning when the bacteria are resistant to carbapenems, a resistance often mediated by specific enzymes called carbapenemases [2,3]. Among Enterobacterales with carbapenem resistance or carbapenemase production (CRE/CPE), *Klebsiella pneumoniae* producing New Delhi Metallo-beta-lactamase-1 (NDM-1) demonstrates a particularly high level of resistance to multiple antibiotics and noted ability to spread, especially in healthcare settings [2,3].

In Germany, national surveillance of CRE/CPE revealed that *K. pneumoniae* was the most frequent species among CPE infections/colonisations in 2022 and in previous years [4]. Since March 2022, Germany has experienced a rapid increase in NDM-1 *K. pneumoniae* [5]. Consequently, NDM-1 was the most frequent carbapenemase among *K. pneumoniae* cases in 2022, whereas previously OXA-48 had predominated [6].

The rise of NDM-1 *K. pneumoniae* coincided with Russia’s invasion of Ukraine in February 2022, which prompted massive westward migration of Ukrainians and medical evacuations involving both military personnel and civilians. Among the European Union/European Economic Area (EU/EEA) countries, Germany received the highest number of Ukrainian refugees [7], and cases from Ukraine have disproportionately contributed to the observed increase in NDM-1 *K. pneumoniae* in Germany since 2022 [5]. Hence, the increase and the association with the war in Ukraine has raised questions about how and where these pathogens were transmitted, e.g. in Ukraine, during transit from Ukraine or in Germany. Understanding such transmission patterns is crucial for informing public health strategies against the pathogen’s spread and infection prevention and control (IPC) measures. 

Within the Integrated Genomic Surveillance (IGS), we conduct surveillance of CPE in Germany, integrating epidemiological data of mandatory notifications with bacterial whole genome sequencing (WGS) information for trend analyses and outbreak detection. Here, we investigated the NDM-1 *K. pneumoniae* cases in Germany from January 2022 to February 2023 as part of IGS to (i) determine the diversity and clonal relatedness of NDM-1 *K. pneumoniae*, (ii) identify and characterise genetic clusters and (iii) delineate transmission patterns using IGS data.

## Methods

### Surveillance system

We extracted data on NDM-1-producing *K. pneumoniae* cases from the IGS that links notifications to data from isolates received by the national reference centre (NRC). 

Colonisation or infection with CRE/CPE (including reduced carbapenem susceptibility) are notifiable to public health authorities in Germany. Within this passive surveillance system, cases are primarily detected through risk-adapted hospital screening, e.g. on admission after hospitalisation in CRE/CPE high prevalence regions, in hospitals with acute outbreaks, before intensive care unit transfer or as part of clinical diagnostics. Notifications include information on location of residence, sex, age, location and dates of hospitalisation within the past 12 months, sampling dates and specimen types, stays abroad within the past 12 months and death.

Laboratories are recommended to submit Enterobacterales isolates suspected to be carbapenemase-producing to the NRC for verification and genotyping. Carbapenemase specification is routinely performed, as described elsewhere [8]. Carbapenemase-producing *Klebsiella pneumoniae* (CP-Kp) isolates were subjected to Illumina WGS.

Sequence information is matched to notifications based on age, residence location, submitting laboratory location, pathogen, carbapenemase types, sampled material, sampling dates, notification dates and isolate reception dates. Non-matching isolates were kept in the dataset.

### Sequencing and cluster identification

Following genomic DNA isolation using the DNeasy Blood and Tissue Kit (Qiagen), libraries were prepared using the Nextera XT DNA Library Preparation Kit (Illumina) and paired-end sequenced on a NextSeq (2 x 151 bp) or MiSeq (2x251 bp) (Illumina). Raw reads were quality-checked using FastQC [9]. Species identification and check for contamination of raw reads were performed using Mash Distance and Mash Screen, respectively [10,11]. Raw reads were de novo assembled using SPAdes (v3.10.1) in the careful mode with default parameters [12,13]. Obtained assemblies were assessed using QUAST [14].Assemblies were analysed using multilocus sequence typing (MLST) and core genome (cg)MLST based on the *K. pneumoniae* sensu lato scheme (2,358 loci) as implemented in the SeqSphere + software version 8.4.1 (Ridom). Following cgMLST, we selected ‘major sequence type (ST)’ groups, i.e. ST for which there were at least 10 isolates. Isolates of these ST were subjected to separate single nucleotide variant (SNV)-based analyses using its function integrated in the SeqSphere + software. We defined ‘clusters’ as groups of isolates showing ≤ 12 SNV differences within genes of the core genome (cgSNV), as previously described [5,15]. The cut-off was derived by identifying a correlate to the criteria defined by Tenover et al. [15]. In brief, on a selection of isolates, *X*baI-macrorestriction and pulsed field gel electrophoresis (PFGE) were performed. Identical banding patterns indicative of clonality corresponded to a maximum of 12 SNV differences that we thus chose as a cut-off to define clusters. [5] ‘Cluster cases’, i.e. cases with clustering isolate sequences, were considered part of a transmission chain. Allelic cgSNV differences between isolates were used to construct neighbour-joining trees per major ST with metadata annotated using Interactive Tree of Life (iTOL) version 6.5.7 [16].To assess clustering with cases of NDM-1 *K. pneumoniae* ST147 cases that were detected across Europe in a plasmid-derived cluster and were linked to spread in Ukraine, we downloaded Illumina whole genome sequencing data [17] and added them to the cgSNV analysis. We excluded those sampled in Lithuania and Latvia where spread was attributed to in-country outbreaks.

### Epidemiological analysis and case definition

We defined ‘cases’ as individuals colonised or infected with NDM-1-producing *K. pneumoniae* confirmed by the NRC based on isolates received from 1 January 2022 to 28 February 2023 (424 days). Duplicate isolates (same species with the same carbapenemases from the same person) were excluded.

Case location was defined as the mid-point of the district of the submitting hospital or, if missing, patient residence or laboratory. We defined ‘case distance’ as the maximum direct distance between cases in one cluster. The ‘case occurrence’ date was defined as the earliest date of sampling, symptom onset, notification and isolate reception at the NRC. Because cases were included in the analysis based on isolate reception at the NRC, dates of case occurrence could be slightly earlier than the inclusion period beginning on 1 January 2022. Cluster durations were calculated as the period between the occurrence of cluster cases. We grouped clusters into ‘exposure location categories’, i.e. ‘no exposure abroad’ if no case had a documented stay abroad within the 12 months prior to detection in Germany, ‘exposure in Ukraine’ if at least one case had a prior stay in Ukraine, ‘other exposure abroad’ if at least one case had a prior stay abroad elsewhere, and ‘missing’ if no isolates could be matched to notifications.

### Cluster follow-up

For 13 clusters with sufficient case information for back-tracing, we conducted a more in-depth cluster follow-up in September 2022 with the cases detected and data available at that timepoint. We contacted the regional and local public health authorities in Germany with a questionnaire requesting more information on cluster cases. The questionnaire collated data on hospital stays in Germany within the 3 months prior to detection, regions in Ukraine before transit to Germany (or origin in Ukraine, if missing), and whether the case was Ukrainian military personnel. We anonymised locations in Ukraine to avoid disclosure of sensitive information. We described the distribution of these variables among cases per cluster and analysed timelines of hospital stays in Germany. Case pairs with stays in the same hospital were identified. A potentially nosocomial transmission was considered if positive sampling occurred 3 days post-admission for one of the cases in a case pair with an overlapping hospital stay. We selected three clusters to illustrate the differing transmission patterns observed.

### Statistical analysis

Descriptive statistics were used to summarise the characteristics of the clusters and cases. Chi-square and Fisher’s exact tests were applied to compare categorical variables, while the Kruskal–Wallis test was used for continuous variables. Statistical significance was set at p < 0.05. Analyses and visualisations were conducted in R, version 4.3.0.

## Results

**Figure 1:**
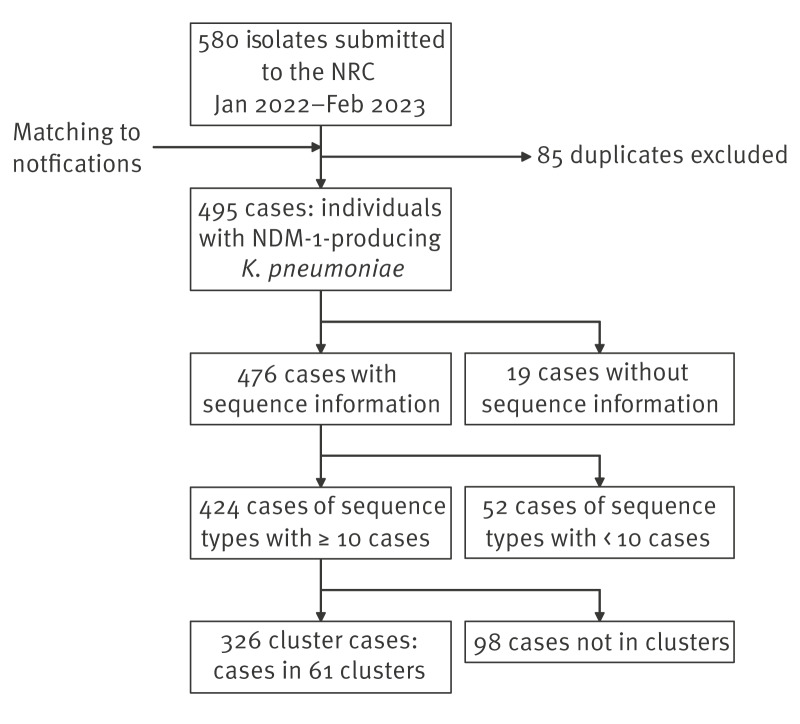
Dataset of NDM-1 *Klebsiella pneumoniae* cases, Germany, January 2022–February 2023 (n = 495)

From January 2022–February 2023, 495 cases with NDM-1 *K. pneumoniae* were detected and confirmed (Figure 1). The NRC received duplicate isolates for 85 individuals, i.e. 580 isolates in total. In 130 of 495 cases (26%), a second carbapenemase was identified, namely OXA-48 (n = 110), OXA-232 (n = 9), KPC-2 (n = 3), KPC-3 (n = 3), OXA-181 (n = 2), OXA-244 (n = 2), and VIM-1 (n = 1).

NRC: national reference centre.

Colonised or infected individuals were included. We obtained pathogen sequences for 476 cases of which 424 occurred among major ST. Among these, 326 were part of clusters.

**Figure 2:**
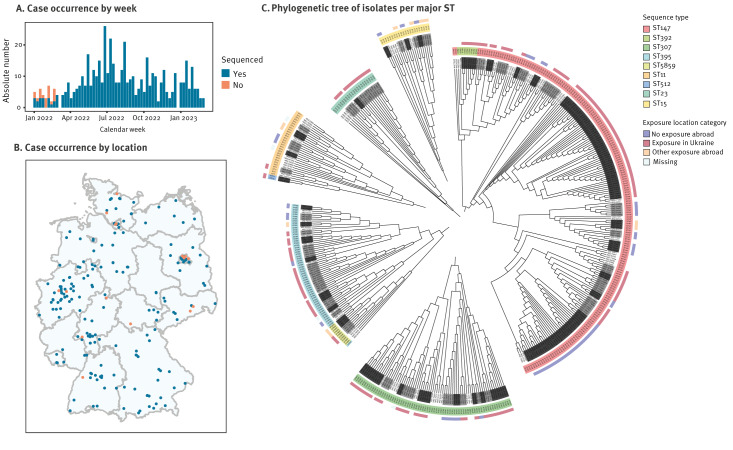
NDM-1-producing *Klebsiella pneumoniae* cases (n = 495) by week and case location and phylogenetic analysis of isolates of major sequence types (n = 424), Germany, January 2022–February 2023

Overall, isolates from 476 of 495 (96%) cases were sequenced (Figure 1, Figure 2A and 2B). Of these, 424 (89%) belonged to major ST, i.e. ST present among at least 10 cases (Figure 2C). These comprised nine different ST (those grouping together phylogenetically are concatenated with a ‘/’): ST147/392 (n = 207 cases, 43%), ST307 (n = 73, 15%), ST395/5859 (n = 69, 14%), ST11/512 (n = 31, 7%), ST15 (n = 22, 5%), and ST23 (n = 22, 5%).

Case occurrence (n = 495) by calendar week (Panel A) and by location (Panel B), colour-coded by availability of pathogen sequences. Phylogenetic trees (Panel C) of sequenced isolates (n = 424) per major ST, highlighting clusters in grey shades (light and dark grey alternating to distinguish adjacent clusters, first inner ring) and by ST (second ring). Clusters are labelled by exposure location categories (third, outer ring).

**Figure 3:**
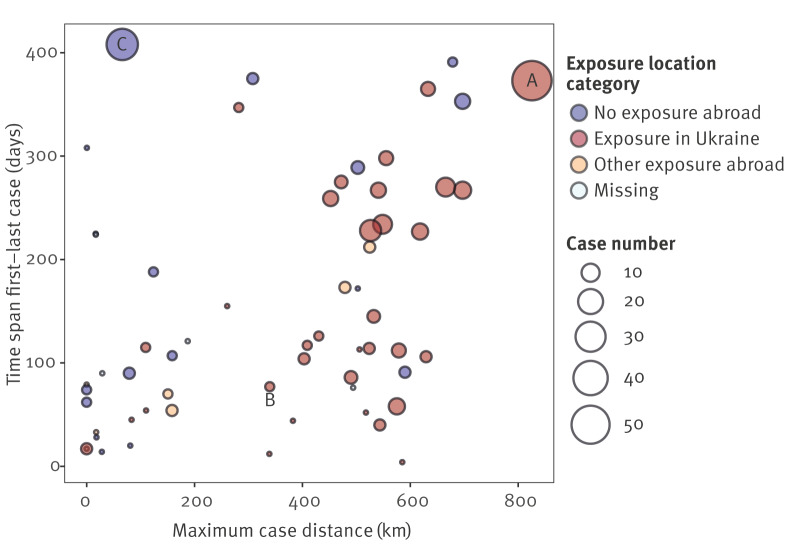
Geographic spread and duration of NDM-1-producing *Klebsiella pneumoniae* clusters by exposure location, Germany, January 2022–February 2023 (n = 61)

Among these 424 cases from major ST, 326 (77%) were part of 61 clusters in total. These clusters varied in size, containing 2–54 cluster cases (Figure 3). The median size was three cases. A third of the clusters (n = 21) consisted of two cases. Five clusters were larger than 10 cases.

**Figure 4:**
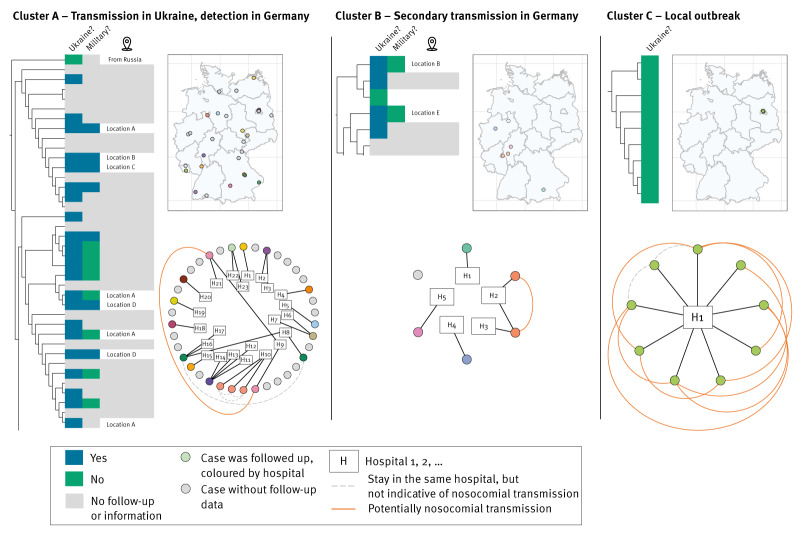
Clusters of NDM-1 *Klebsiella pneumoniae* after cluster follow-up exemplifying different transmission patterns, Germany, January 2022–February 2023 (n = 3)

Heterogeneity in time as cluster duration (y-axis), in space as case distance (x-axis), in number of cases per cluster (bubble size), and in exposure location category (colour). Letters indicate the clusters selected for cluster follow-up with scenarios A (transmission in Ukraine), B (secondary transmission in Germany), and C (local outbreak), also see Figure 4.

### Cluster characteristics

Of the 61 clusters, 17 (28%) had no cases with a documented stay abroad 12 months before detection, and 33 (54%) had cases with exposure in Ukraine (Figure 2C, Figure 3). These clusters comprised 84 and 213 cases, respectively. Seven (11%, comprising 21 cases overall) clusters contained cases with other exposure abroad, i.e. reported travel to or from Albania, Azerbaijan, Iran, Serbia, Southern European countries (unspecified), Spain and Syria. For four (7%) clusters comprising eight cases, information on exposure location category was missing.

Clusters did not differ in size depending on exposure location category. Carbapenemases other than NDM-1 or NDM-1 + OXA-48 occurred only in clusters with cases exposed abroad. ST23 only occurred in clusters with exposure in Ukraine, ST307 in clusters with exposure in Ukraine or other exposure abroad, and ST15 in clusters with other exposure abroad or no exposure abroad. Clusters belonging to ST11/512, ST147/392, and ST395/5859 occurred in all exposure location categories.

The earliest sampling date of a cluster case was 1 November 2021. Cluster cases continued to occur until the end of the observation period, i.e. the latest case on 14 February 2023. Cluster durations were 4–408 days, with a median of 114 days. Clusters with no exposure abroad had the longest median duration of 172 days, followed by clusters with exposure in Ukraine (115 days) and with other exposure abroad (70 days, p > 0.05).

Cluster cases occurred in all 16 German federal states. Across clusters, the median case distance was 410 km, ranging from 0 to 826 km. Clusters with exposure in Ukraine had the highest case distance (p < 0.01), with a median of 518 km, compared with 151 km for clusters with other exposure abroad and 81 km for clusters without exposure abroad. When plotting clusters by duration and case distance (Figure 3), clusters with exposure abroad tended to occupy the plot’s area on the lower right from the diagonal, i.e. a wider case distance at shorter cluster duration. Clusters with no exposure abroad tended to be on the upper left from the plot’s diagonal, i.e. longer cluster duration and spatially confined.

By the exposure location category, cluster cases did not significantly differ by sex (Table). Cases in clusters with exposure in Ukraine were significantly younger on average (p < 0.001), with a median age of 45 years compared with 64 years otherwise. Cluster cases did not differ significantly by their proportion of infections vs colonisations, positive screening samples, and death among infected cases. Infected cases in clusters with no exposure abroad more frequently had a positive blood sample (p < 0.01). Those in clusters with exposure in Ukraine more frequently had positive wound samples (p < 0.001) and less frequently detections in urine (p < 0.05).

**Table:** Characteristics of cluster cases of NDM-1 *Klebsiella pneumoniae* by exposure location category, Germany, January 2022–February 2023 (n = 318)

Variable	Cluster exposure location category						p
	No exposure abroad (n = 84)		Exposure in Ukraine (n = 213)		Other exposure abroad (n = 21)		
	n	%	n	%	n	%	
Sex							
Female	10	20	43	31	5	29	NS
Male	41	80	94	69	12	71	
Missing	33		76		4		
Age							
Median years (IQR)	64 (52–76)		45 (31–60)		64 (53–73)		< 0.001
Missing	0		2		0		
Infection/colonisation status							
Infected	52	64	118	56	11	55	NS
Colonised	29	36	91	44	9	45	
Missing	3		4		1		
Detection in blood sample							
Yes	16	19	12	6	1	5	< 0.01
Not indicated	68	81	201	94	20	95	
Detection in wound sample							
Yes	9	11	70	33	0	0	< 0.001
Not indicated	75	89	143	67	21	100	
Detection in urine sample							
Yes	28	33	41	19	8	38	< 0.05
Not indicated	56	67	172	81	13	62	
Detection in screening sample							
Yes	38	45	120	56	12	57	NS
Not indicated	46	55	93	44	9	43	
Death among infected							
Yes	5	16	5	6	0	0	NS
No	27	84	73	94	9	100	
Missing	20		40		2		

NS: not significant.

Chi-square and Fisher’s exact tests were applied to compare categorical variables, while the Kruskal–Wallis test was used for continuous variables. Statistical significance was set at p < 0.05. Characteristics and proportions are described for cases for which the respective information was available. Because sampling material is recorded by a checkbox for ‘yes’ without an explicit option for ‘no’, the alternative is labelled as ‘not indicated’ here. Cases were classified as to their cluster’s exposure location category. Four clusters without matched cases (n = 8) were excluded.

### Cluster follow-up

At the time of cluster follow-up, Cluster A contained 38 cases (Figure 4). Of the 23 cases with available information on prior stays abroad, 22 had come from Ukraine and one from Russia. Seven of the 15 cases from Ukraine with available information were Ukrainian military personnel. The locations in Ukraine before transit to Germany were documented for eight cases. Among these, four cases were from the same location, two other cases shared a different location and two additional cases were from unique locations. Cases were detected across Germany from April until September 2022, i.e. until cluster follow-up. Hospital names and dates of hospital stays were available for 18 cases, in 23 hospitals in Germany overall. Two cases from Ukraine were in the same hospital (H8) but not at the same time. A family of three Ukrainian cases tested positive at screening on admission on the same day in one hospital (H10). Two cases from Ukraine were in the same hospital (H9) with overlapping stays on different wards. One case tested positive on admission, the other on day 8 of the hospital stay. Both had positive screening and wound samples.

Each panel for Clusters A (left), B (middle), and C (right) shows the phylogenetic tree of isolates from cluster cases, a map of the case location, and a hospital stay network. The phylogenetic tree is appended by information whether cases had a prior stay in or originated from Ukraine, the location in Ukraine (anonymised), and whether the case was Ukrainian military personnel. The hospital stay network contains all cluster cases as dots in a circle, linked to hospitals (enumerated separately per cluster) based on their hospital stay history up to 3 months before detection. Cases share a colour if they had a stay in the same hospital (or grey colour if the hospitalisation history was not available). If sampling and admission dates fulfilled the criteria for a potentially nosocomial transmission, an orange line links the cases (dashed and grey, otherwise, i.e. same hospital but dates do not suggest nosocomial transmission or are missing).

We repeated the cgSNV analysis with additional 35 NDM-1 *K. pneumoniae* ST147 cases who were detected across Europe in a plasmid-derived cluster and were linked to spread in Ukraine. Overall, 34 of these 35 cases clustered with the cases in Cluster A. The phylogenetic tree including these 35 cases is provided as Supplementary Figure S1.

Cluster B contained six cases at the time of cluster follow-up. Of the five cases with available information, four had come from Ukraine. For two of these, information on locations of prior exposure in Ukraine was available, but the locations differed. One case had no link to Ukraine. Cases were detected between May and August 2022 in four federal states. Two cases had an overlapping stay in one hospital in adjacent rooms over 25 days. The first case was from Ukraine and had a positive screening sample on day 2 of hospitalisation. The second case was not from Ukraine and had a positive swab in a different hospital after transfer.

Regarding Cluster C, nine cases were captured between November 2021 and August 2022. None of the cases had been in Ukraine, and all were hospitalised in one hospital. All cases could be paired with at least one other case in terms of simultaneous hospital stays and a first positive sample at least 3 days post-admission, except for one case. The outbreak investigation that continued beyond the follow-up period reported here identified further cluster cases in the hospital.

## Discussion

Following the increase of NDM-1 *K. pneumoniae* cases in Germany [5], we investigated cases and clusters in Germany to delineate heterogeneity, extent of clustering and underlying transmission patterns. A quarter of cases had additional carbapenemases besides NDM-1, mostly OXA-48. Similar carbapenemase frequencies had been described for CPE in Germany previously [18]. We detected the rarer non-NDM-1 + OXA-48 combinations only in clusters with exposure abroad, suggesting sporadic introductions.

We detected some ST11/512, ST15, ST147/392, ST307, and ST395/5859 clusters with no exposure abroad, hinting at endemicity in Germany. ST11/512, ST15, and ST147/392 were also among the most abundant ST of CP-Kp in a study across multiple hospitals in Germany between 2008 and 2014 and in a review from 2020 [2,19]. Further studies will need to verify whether the ST have reached (supra)regional endemicity in Germany – as suggested for ST147, given that even the outbreak investigation behind Cluster C identified no link to foreign exposure.

With three quarters of cases belonging to clusters, an unanticipated high fraction of cases could be attributed to chains of transmission. Clusters occurred in high numbers, throughout the observation period, and country-wide. Many clusters contained no cases with documented exposure abroad, hinting at a high degree of parallel chains of transmission within Germany.

Three transmission scenarios emerged: transmission abroad, onward transmission in Germany, and local outbreaks in Germany. Half of all identified clusters had cases with exposure in Ukraine. The ST of these clusters corresponded well to recent studies restricted to Ukrainian patients [20,21]. In a survey in Ukrainian military hospitals in 2022–2023, CP-Kp mostly harboured *bla*_NDM-1_ or *bla*_OXA-48_ and matched in terms of ST [22]. Clusters with exposure in Ukraine were protracted in time and more geographically dispersed across Germany. Cases were younger and more frequently had wound infections. These findings would align with transmission in Ukraine or during transit to Germany among soldiers or civilians affected by the war and medically evacuated. Cluster A exemplifies this scenario: All cases with available information were from Ukraine (or Russia), detected across Germany, and only one case pair had hospital stays and sampling dates in line with nosocomial transmission in Germany. These observations correspond with previous studies on the international spread of CRE/CPE from Ukraine [23]. They are also in line with reports of high CRE/CPE incidence in Ukraine before and since the Russian invasion, and the exacerbation of antibiotic resistance in *K. pneumoniae* by the war [22,24,25]. The hypothesis of transmission in Ukraine before detection in Germany is corroborated by the fact that almost all sequences of cases collected for a European wide analysis and that were linked to spread in Ukraine [17] belong to Cluster A in our investigation, which was also linked to Ukraine.

Cluster B provided an example of onward nosocomial transmission from an imported case, as has been described earlier [26]. Hence, robust IPC in the receiving countries and hospitals remain important, including on-admission screening in case of prior healthcare in endemic countries.

In Cluster C, all cases were linked to one hospital and unrelated to exposure abroad, potentially resembling a nosocomial outbreak in the hospital or transmission in its catchment area. Consequently, the cluster illustrates that CP-Kp transmission in Germany can lead to large, protracted outbreaks. Local spread before the nosocomial outbreak was also hypothesised in a previous outbreak investigation [27]. This underpins the need to implement and assess targeted IPC measures such as admission screening based on additional risk factors, and comprehensive microbiological screening when nosocomial outbreaks are suspected.

Protracted and/or widespread clusters might resemble outbreaks that are difficult to detect, trace, and contain without surveillance routinely integrating comprehensive and nationwide genotyping information. WGS-based typing subdivided ‘NDM-1 *K. pneumoniae*’ into numerous sequence types and clusters. Consequently, we could search for transmission patterns and outbreak signals in smaller data fractions. Hence, IGS promises to increase the resolution of epidemiological surveillance beyond the level of other typing methods, such as pulse field-gel electrophoresis [28]. When describing clusters by person, place and time, plausible transmission patterns emerged, e.g. clusters with cases exposed abroad tended to be more geographically dispersed in Germany. In cluster follow-up, such patterns were confirmed: cases in the dispersed Cluster A all originated from Ukraine or Russia with little to no overlap in hospitalisation histories, and vice versa for cases in cluster C. The correspondence of epidemiological description and cluster information is promising for using cluster information as an adequate signal for outbreak detection. Future studies should be designed to assess how many clusters truly reflect chains of transmission, which would support the potential of IGS in Germany to strengthen surveillance, epidemiologic inference of transmission patterns, and public health action.

This analysis benefits from a large dataset of cases detected in Germany. However, we recognise the following limitations. Firstly, case ascertainment was potentially biased: patient screening, isolate submission, or more detailed case documentation might occur more often for cases from Ukraine, other high-risk patient groups, or upon suspected local transmissions. This could have increased the proportion of cases in clusters and of clusters linked to Ukraine in this study. Secondly, incomplete metadata and matching may have limited the analysis. Thirdly, WGS-based clustering was used as a proxy for chains of transmission. Since there is no established cut-off, we based it on a correspondence to the Tenover criterion for clonality (see Methods). Varying mutation rates among bacterial lineages could imply decreased sensitivity and specificity of the cut-off. However, cases in clusters shared epidemiological links, e.g. exposure abroad in Clusters A and B and hospitalisation histories in Cluster C, arguing against overly low specificity. Fourthly, we assumed an epidemiologic link between cluster cases when these were hospitalised simultaneously in the same hospital. Although a strong signal, this does not necessarily reflect a nosocomial outbreak in that hospital. It could also resemble previous transmission in other hospitals (if an outbreak was not detected there) or in outpatient settings (where almost no testing occurs). Finally, collated locations in Ukraine before transfer to Germany did not yield a match across most cases. That no transmission hotspots in Ukraine were identified might reflect incomplete information. Investigating transmission locations in Ukraine or during transit should provide insights for targeting IPC measures and outbreak control.

## Conclusions

Many NDM-1-producing *K. pneumoniae* cases in Germany from January 2022 to February 2023 could be assigned to clusters, i.e. putative chains of transmission. Many clusters likely stemmed from transmission in Ukraine, arguing for increased IPC support in Ukraine. The occurrence of multiple clusters across Germany, with or without exposure abroad, and the high proportion of cases in clusters underscores the importance of IPC and public health action. They suggest a considerable potential for the IGS to detect clusters, localise transmission, and guide prevention measures. Comprehensive sharing of isolates and/or sequences and all relevant information for notifiable cases is important for maximum depth of analysis. Joint efforts of public health authorities, healthcare providers, and decisionmakers are needed to support a comprehensive IGS to mitigate the spread of antibiotic-resistant pathogens.

## Data Availability

Anonymised data and data dictionaries can be shared upon reasonable request to the corresponding author. Sequence data were deposited in the NCBI's Sequence Read Archive (SRA) related to the BioProjects PRJEB58018 and PRJNA1268013.

## Supplementary Data

549000 Supplement

## References

[1] Murray CJL Ikuta KS Sharara F Swetschinski L Robles Aguilar G Gray A Global burden of bacterial antimicrobial resistance in 2019: a systematic analysis. Lancet. 2022;399(10325):629-55. 10.1016/S0140-6736(21)02724-0 35065702 PMC8841637

[2] Wyres KL Lam MMC Holt KE. Population genomics of Klebsiella pneumoniae. Nat Rev Microbiol. 2020;18(6):344-59. 10.1038/s41579-019-0315-1 32055025

[3] European Centre for Disease Prevention and Control (ECDC). Carbapenem-resistant Enterobacteriaceae, second update. Stockholm: ECDC; 2019. Available from: https://www.ecdc.europa.eu/en/publications-data/carbapenem-resistant-enterobacteriaceae-second-update

[4] Robert Koch-Institut (RKI). Infektionsepidemiologisches Jahrbuch meldepflichtiger Krankheiten für 2022. [Infection Epidemiology Yearbook of Notifiable Diseases for 2022]. Berlin: RKI; 2024. Available from: https://edoc.rki.de/handle/176904/11825.2

[5] Sandfort M Hans JB Fischer MA Reichert F Cremanns M Eisfeld J Increase in NDM-1 and NDM-1/OXA-48-producing Klebsiella pneumoniae in Germany associated with the war in Ukraine, 2022. Euro Surveill. 2022;27(50):2200926. 10.2807/1560-7917.ES.2022.27.50.2200926 36695468 PMC9808319

[6] Pfennigwerth N Cremanns M Eisfeld J Hans J Anders A Gatermann S. Bericht des Nationalen Referenzzentrums für gramnegative Krankenhauserreger – Zeitraum 1. Januar 2022 bis 31. Dezember 2022. Epid Bull. 2023;27:3-10. German. 10.25646/11589

[7] UNHCR. Estimated number of refugees from Ukraine recorded in Europe and Asia since February 2022 as of July 2024, by selected country. Hamburg: Statista; 7 Feb 2025. Available from: https://www.statista.com/statistics/1312584/ukrainian-refugees-by-country

[8] Pfennigwerth N Gatermann SG Körber-Irrgang B Hönings R. Phenotypic detection and differentiation of carbapenemase classes including OXA-48-like enzymes in Enterobacterales and Pseudomonas aeruginosa by a highly specialized micronaut-S microdilution assay. J Clin Microbiol. 2020;58(11):e00171-20. 10.1128/JCM.00171-20 32878951 PMC7587086

[9] Andrews S. FastQC: a quality control tool for high throughput sequence data. Cambridge: Github. [Accessed: 7 Feb 2025]. Available from: https://github.com/s-andrews

[10] Ondov BD Starrett GJ Sappington A Kostic A Koren S Buck CB Mash Screen: high-throughput sequence containment estimation for genome discovery. Genome Biol. 2019;20(1):232. 10.1186/s13059-019-1841-x 31690338 PMC6833257

[11] Ondov BD Treangen TJ Melsted P Mallonee AB Bergman NH Koren S Mash: fast genome and metagenome distance estimation using MinHash. Genome Biol. 2016;17(1):132. 10.1186/s13059-016-0997-x 27323842 PMC4915045

[12] Bankevich A Nurk S Antipov D Gurevich AA Dvorkin M Kulikov AS SPAdes: a new genome assembly algorithm and its applications to single-cell sequencing. J Comput Biol. 2012;19(5):455-77. 10.1089/cmb.2012.0021 22506599 PMC3342519

[13] Prjibelski A Antipov D Meleshko D Lapidus A Korobeynikov A. Using SPAdes de novo assembler. Curr Protoc Bioinformatics. 2020;70(1):e102. 10.1002/cpbi.102 32559359

[14] Gurevich A Saveliev V Vyahhi N Tesler G. QUAST: quality assessment tool for genome assemblies. Bioinformatics. 2013;29(8):1072-5. 10.1093/bioinformatics/btt086 23422339 PMC3624806

[15] Tenover FC Arbeit RD Goering RV Mickelsen PA Murray BE Persing DH Interpreting chromosomal DNA restriction patterns produced by pulsed-field gel electrophoresis: criteria for bacterial strain typing. J Clin Microbiol. 1995;33(9):2233-9. 10.1128/jcm.33.9.2233-2239.1995 7494007 PMC228385

[16] Letunic I Bork P. Interactive Tree Of Life (iTOL) v5: an online tool for phylogenetic tree display and annotation. Nucleic Acids Res. 2021;49(W1):W293-6. 10.1093/nar/gkab301 33885785 PMC8265157

[17] Linkevicius M Alm E Roer L Svartström O Dada-Olorunwa M Räisänen K Cross-border spread of a mosaic resistance (OXA-48) and virulence (aerobactin) plasmid in Klebsiella pneumoniae: a European Antimicrobial Resistance Genes Surveillance Network investigation, Europe, February 2019 to October 2024. Euro Surveill. 2025;30(27):2500439. 10.2807/1560-7917.ES.2025.30.27.2500439 40642770 PMC12262110

[18] Pfennigwerth N Schauer J. Bericht des Nationalen Referenzzentrums für gramnegative Krankenhauserreger – Zeitraum 1. Januar 2021 bis 31. Dezember 2021.[Report of the National Reference Center for Gram-negative Hospital Pathogens Period January 1, 2021 to December 31, 2021]. Epid Bull. 2022;19:3-9. German. 10.25646/10034

[19] Becker L Kaase M Pfeifer Y Fuchs S Reuss A von Laer A Genome-based analysis of Carbapenemase-producing Klebsiella pneumoniae isolates from German hospital patients, 2008-2014. Antimicrob Resist Infect Control. 2018;7(1):62. 10.1186/s13756-018-0352-y 29744043 PMC5930415

[20] Zwittink RD Wielders CC Notermans DW Verkaik NJ Schoffelen AF Witteveen S Multidrug-resistant organisms in patients from Ukraine in the Netherlands, March to August 2022. Euro Surveill. 2022;27(50):2200896. 10.2807/1560-7917.ES.2022.27.50.2200896 36695467 PMC9808315

[21] Schultze T Hogardt M Velázquez ES Hack D Besier S Wichelhaus TA Molecular surveillance of multidrug-resistant Gram-negative bacteria in Ukrainian patients, Germany, March to June 2022. Euro Surveill. 2023;28(1):2200850. 10.2807/1560-7917.ES.2023.28.1.2200850 36695452 PMC9817211

[22] Kovalchuk V Kondratiuk V McGann P Jones BT Fomina N Nazarchuk O Temporal evolution of bacterial species and their antimicrobial resistance characteristics in wound infections of war-related injuries in Ukraine from 2014 to 2023. J Hosp Infect. 2024;152:99-104. 10.1016/j.jhin.2024.06.011 38997008

[23] Witteveen S Hans JB Izdebski R Hasman H Samuelsen Ø Dortet L Dissemination of extensively drug-resistant NDM-producing Providencia stuartii in Europe linked to patients transferred from Ukraine, March 2022 to March 2023. Euro Surveill. 2024;29(23):2300616. 10.2807/1560-7917.ES.2024.29.23.2300616 38847120 PMC11158010

[24] Kondratiuk V Jones BT Kovalchuk V Kovalenko I Ganiuk V Kondratiuk O Phenotypic and genotypic characterization of antibiotic resistance in military hospital-associated bacteria from war injuries in the Eastern Ukraine conflict between 2014 and 2020. J Hosp Infect. 2021;112:69-76. 10.1016/j.jhin.2021.03.020 33789157

[25] Salmanov A Shchehlov D Svyrydiuk O Bortnik I Mamonova M Korniyenko S Epidemiology of healthcare-associated infections and mechanisms of antimicrobial resistance of responsible pathogens in Ukraine: a multicentre study. J Hosp Infect. 2023;131:129-38. 10.1016/j.jhin.2022.10.007 36306892

[26] Smit WL Wunderink HF Kluytmans JAJW Tissing WJE van Dijkhuizen EHP Loeffen YGT Nosocomial transmission of NDM-1-containing Klebsiella pneumoniae ST147 in a Dutch pediatric oncology center associated with patients from Ukraine. BMC Infect Dis. 2024;24(1):1460. 10.1186/s12879-024-10368-2 39716112 PMC11667838

[27] Haller S Kramer R Becker K Bohnert JA Eckmanns T Hans JB Extensively drug-resistant Klebsiella pneumoniae ST307 outbreak, north-eastern Germany, June to October 2019. Euro Surveill. 2019;24(50):1900734. 10.2807/1560-7917.ES.2019.24.50.1900734 31847948 PMC6918589

[28] Gona F Comandatore F Battaglia S Piazza A Trovato A Lorenzin G Comparison of core-genome MLST, coreSNP and PFGE methods for Klebsiella pneumoniae cluster analysis. Microb Genom. 2020;6(4):e000347. 10.1099/mgen.0.000347 32149598 PMC7276701

