# Supplementary material for: International spread or local outbreak? Epidemiologic analyses of transmission patterns of NDM-1-producing Klebsiella pneumoniae based on genomic surveillance data, Germany, January 2022 to February 2023
Source: Euro Surveill. 2026 Jan 8;31(1):2500378. doi: 10.2807/1560-7917.ES.2026.31.1.2500378 (PMC12862290; doi:10.2807/1560-7917.ES.2026.31.1.2500378)
Supplement: Supplement [file 25-00378_SANDFORT_Supplementary_Figure.pdf]

This supplementary material is hosted by Eurosurveillance as supporting information alongside the article "International spread or local outbreak? Epidemiologic analyses of transmission patterns of NDM-1 producing Klebsiella pneumoniae based on genomic surveillance data, Germany, Jan 2022-Feb 2023", on behalf of the authors, who remain responsible for the accuracy and appropriateness of the content. The same standards for ethics, copyright, attributions and permissions as for the article apply. Supplements are not edited by Eurosurveillance and the journal is not responsible for the maintenance of any links or email addresses provided therein.

Clustering of NDM-1 K. pneumoniae ST147 cases detected in Germany or across Europe

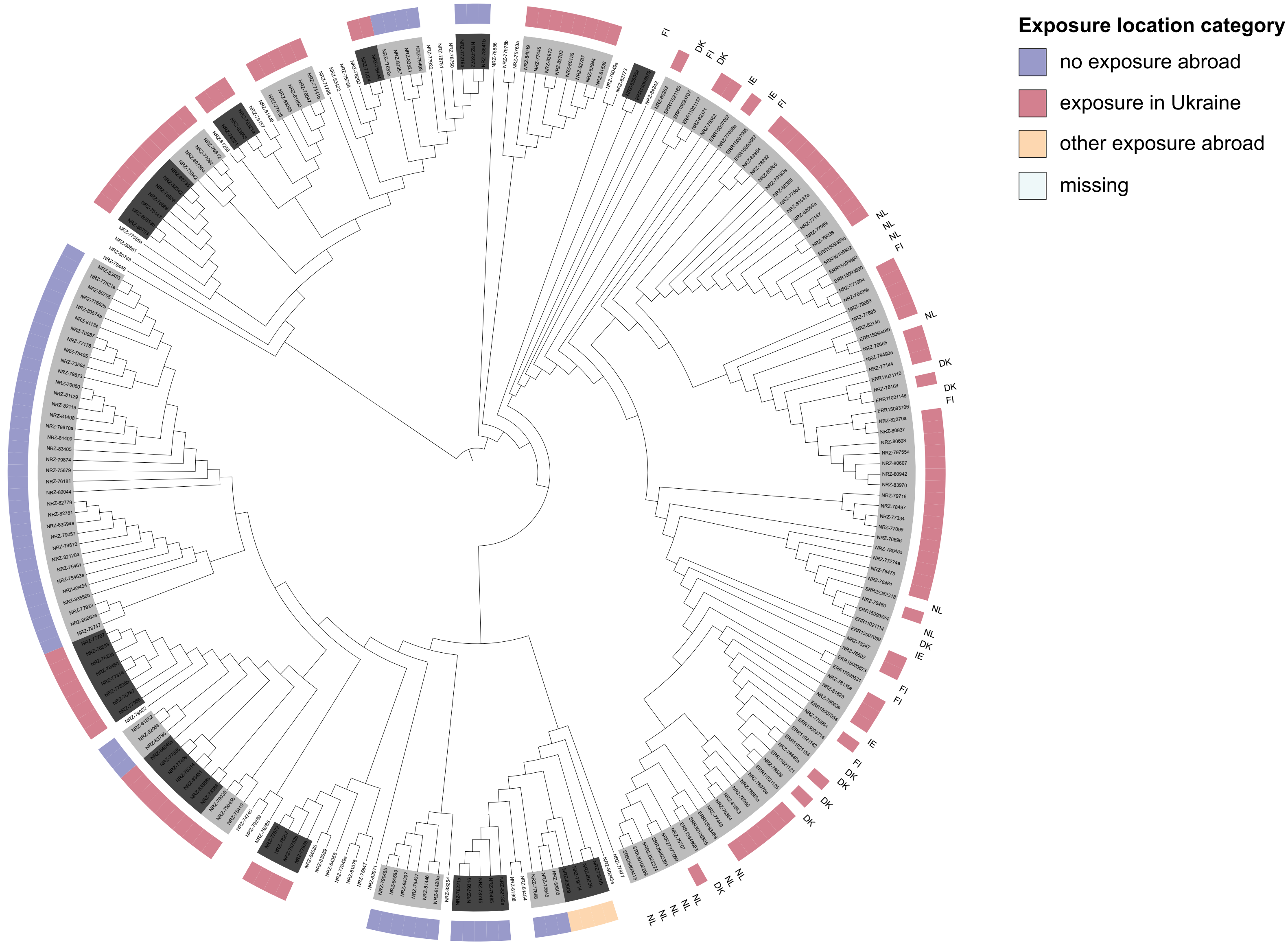

Supplementary Figure S1 NDM-1-producing *Klebsiella pneumoniae* ST147 cases in Germany, 01/2022-02/2023 (n=208) and Europe (n=35). Phylogenetic tree highlighting clusters in grey shades (light and dark grey alternating to distinguish adjacent clusters, 1st ring) and by exposure location categories (2nd ring). Cases that were detected across Europe and linked to spread in Ukraine are labelled by country of detection (3rd ring).
